# Pediatric Oncology Providers’ HPV Vaccine Knowledge, Attitude, Self-Efficacy, and Practice after Communication Training: A Comparison with a National Survey

**DOI:** 10.3390/vaccines12091060

**Published:** 2024-09-17

**Authors:** Rejane A. Teixeira, Allison Grimes, Leanne Embry, Christine Aguilar, L. Aubree Shay

**Affiliations:** 1 UT Health Houston School of Public Health in San Antonio, Department of Health Promotion and Behavioral Sciences, San Antonio, TX 78216, USA; 2 UT Health San Antonio, Department of Pediatrics, Division of Pediatric Hematology and Oncology, San Antonio, TX 78216, USAembryl@uthscsa.edu (L.E.);; 3 Greehey Children’s Cancer Research Institute, San Antonio, TX 78216, USA

**Keywords:** childhood cancer survivors, knowledge, attitude, self-efficacy, vaccine practices, human papillomavirus (HPV)

## Abstract

Background/Objectives: Human papillomavirus (HPV) vaccinations prevent HPV infection and related cancers. Despite being at higher risk of secondary cancers linked to HPV, childhood cancer survivors (CCS) are undervaccinated. This study aimed to compare pediatric oncology providers’ knowledge, attitudes, self-efficacy, and practices regarding HPV vaccination among those who participated in a multilevel educational HPV vaccine program with those of a national sample of oncology providers. Methods: Between February and March 2023, 39 providers from five pediatric oncology clinics in Texas completed online surveys, assessing knowledge about CCS risk for HPV-related cancers, attitudes towards the HPV vaccine, and confidence in recommending the vaccine to CCS. The results were compared with a national survey of providers conducted in 2019 (n = 195). Results: The findings showed that providers who participated in our program had greater knowledge of CCS increased risk for HPV-related cancers (96% vs. 38%; *p* < 0.001); greater confidence in discussing and recommending the HPV vaccine (100% vs. 66%, *p* < 0.001) and addressing parental concerns (100% vs. 69%, *p* < 0.001); and a more positive attitude about oncology providers than general pediatricians, recommending (96% vs. 71%; *p* = 0.006) and administering the HPV vaccine to CCS (96% vs. 53%, *p* < 0.001). Conclusion: This study underscores the importance of educating oncology providers about the increased risk of CCS and improving their self-efficacy to recommend the HPV vaccine, promoting vaccination in the oncology setting.

## 1. Introduction

According to estimates by the National Cancer Institute, as of January 2020, there were nearly 496,000 childhood cancer survivors (CCS) in the United States [1]. With survival rates approaching 85% for pediatric cancers, this burgeoning survivorship population faces a multitude of life-threatening late effects, including secondary malignancies, which occur as early as young adulthood in 1 in 10 childhood cancer survivors [2]. Additionally, CCS are uniquely vulnerable to HPV-related second cancers. Recent studies indicated that male CCS are 150% more likely and female CCS are 40% more likely than the general population to develop HPV-related cancers [3]. Specifically, Henderson et al. [4] found that CCS, irrespective of gender, have a four- to eightfold increased risk of oropharyngeal cancer. It is estimated that about 70% of these oropharyngeal malignancies are linked to HPV [5]. Their increased risk of acquiring and maintaining HPV is attributable in part to their cancer treatment and persistent immunosuppression [3]. In recognition of these risks and the fact that childhood cancer survivors have shown an immune response to the HPV vaccine comparable to their peers who have no history of cancer [6], current guidelines recommend HPV vaccination for all age-eligible cancer survivors [7].

Alarmingly, HPV vaccination rates for CCS lag far behind their peers without a history of cancer. A study of 982 CCS treated at five comprehensive cancer treatment facilities in the United States found that just 24% of survivors aged 13 to 26 started HPV vaccination, and only 13.5% completed the three-dose regimen [3]. Furthermore, another study found that CCS who had chemotherapy missed more HPV vaccination chances than those who did not receive chemotherapy [8]. According to these data, up to 85% of CCS lack full series HPV protection, putting them at further risk for infection and future malignancies. Thus, HPV vaccine initiatives targeting this vulnerable population are especially critical, and there is a clear gap in the implementation of guideline-based recommendations.

A number of factors may undermine HPV vaccination uptake among CCS. Previous research has found that CCS who receive a recommendation from their oncology care team are more likely to get vaccinated [9]. However, many CCS do not transition back to a pediatrician or primary care physician (PCP) for preventive care after their cancer treatment ends, instead receiving ongoing care from their oncologist. Because oncologists do not routinely order vaccines, CCS may never be offered the HPV vaccine despite their increased risk for future HPV-related malignancies. Thus, a significant gap in services exists for this high-risk group.

Compounding the problem, most oncologists do not have training in making high-quality HPV vaccine recommendations, and there is often confusion among providers about who is responsible for discussing HPV vaccination [10]. While oncologists often believe that PCPs are responsible for all preventive care, including vaccinations, PCPs report that oncologists should be responsible for providing the HPV vaccines, given its association with cancer [11]. Prior to our program, no previous training was specifically developed for oncology providers, leaving a void in providing specialized care for this demographic. This lack of targeted training has also contributed to a gap in research about CCS and their specific needs regarding HPV vaccination.

The provider communication training we developed, adapted from the HPV-IQ program [12] and described in more detail below, is a significant stride in addressing a notable gap in research and is specifically tailored to the unique needs of childhood cancer survivors. This program aims to equip oncology providers with the necessary skills and knowledge to effectively recommend the HPV vaccine to this vulnerable group. Furthermore, after-training surveys of providers give valuable insight into opportunities for improvement and implementation challenges. These insights are crucial to keep the training current, effective, and sensitive to the evolving needs of clinicians and CCS. Therefore, this initiative enhances immediate patient care and contributes to the broader research landscape concerning HPV vaccination in CCS.

The objective of this study was to compare the knowledge, attitudes, and self-efficacy to recommend the HPV vaccine among providers in our multilevel educational HPV Vaccine program with those of a national survey of pediatric oncology providers.

## 2. Materials and Methods

### 2.1. Childhood Cancer Survivor HPV Prevention Program

A multilevel intervention aimed at increasing the HPV vaccination rates among CCS in Texas was developed using the American Cancer Society’s “Steps for Increasing HPV Vaccination in Practice” plan [13]. The intervention, called Childhood Cancer Survivor HPV Prevention Program (henceforth, Survivor HPV Program), was implemented at five pediatric oncology programs across Texas that are part of the Texas Pediatric Minority Underserved (TPMU) NCI Community Oncology Research Program (NCORP) [14,15]. From 2019 to 2023, we conducted a yearly continuing education training intended for pediatric oncology providers and staff, as well as practice-level changes to foster an HPV-vaccine-friendly culture and facilitate the monitoring of CCS eligibility for HPV vaccination and on-site vaccine administration. This included working with pharmacy and clinic administration as needed to ensure that vaccines could be stored and administered within the CCS clinics.

In 2019, a 90 min training was conducted on-site for each participating oncology clinic and was offered to both physician and nonphysician staff involved in clinical care for off-therapy CCS (e.g., doctors, nurses, medical assistants, pharmacists, and psychosocial team members). The training included 2 parts: (1) educational information on HPV from the Centers for Disease Control (CDC), risks of HPV-driven second cancers in CCS and HPV vaccination barriers in this population, and (2) communication skills training adapted to the CCS context from HPV-IQ, a validated training tool to equip providers to offer strong HPV vaccine recommendations [16,17]. From 2020 to 2023, annual refresher courses were conducted online, lasting one hour. These annual refresher courses included the same materials and updated responses to frequently asked questions about HPV vaccination.

### 2.2. Study Design and Participants

For this study, we compared attitudes, knowledge, and self-efficacy among two sets of oncology providers: those who practiced at one of the five sites included in the Survivor HPV Program described above and a national sample of pediatric oncologists surveyed by our research team in 2019. In both instances, participants completed online surveys to assess their knowledge about CCS being at higher risk for developing HPV-related secondary malignancies, attitudes towards the HPV vaccine, and self-efficacy in recommending the HPV vaccine to CCS. They also answered questions about their current vaccination practices, including discussing and recommending the HPV vaccine as part of their routine practice. The recruitment procedures for each sample of providers are described in more detail below.

### 2.3. National Survey

We purchased a dataset containing the contact details of 1614 practicing pediatric oncology specialists in the U.S. from IQVIA, a widely used medical marketing database that is updated every six months [18]. From May to August 2019, recruitment emails were sent to a sample of 1285 of those providers. Informed consent elements were included in the survey’s introduction page. Participants received an incentive ($20 Amazon gift card) to complete the survey. This survey study was reviewed and approved by the UTHealth Houston institutional review board (HSC-SPH-19-0275).

### 2.4. Survivor HPV Program Survey

We surveyed Texas pediatric oncology providers who practiced at any of the participating clinics in our Survivor HPV Program during the final year of our project. In February and March 2023, emails were sent to 88 physicians and advanced practice providers in the five clinics affiliated with the TPMU NCORP. Similar to the national survey, the consent form was embedded in the introduction page. No incentives were provided for participation in this survey.

### 2.5. Survey Items Common to Both Groups

Common survey items included in this study were provider self-reported sex, years in practice (0–5 years; 6–10 years; 11–20 years; or 20+ years), and seven items related to HPV vaccination. To measure providers’ knowledge, attitudes, and self-efficacy in discussing and recommending the HPV vaccine to CCS, 5-point Likert scales (ranging from “strongly disagree” to “strongly agree”) were used to indicate their level of agreement with HPV infection and knowledge statements. To measure HPV vaccination practices, we asked providers to rate how often they discussed or recommended the HPV vaccine for age-eligible cancer survivors on a 5-point Likert scale ranging from “always” to “never”. All common survey items are available in Appendix A.

### 2.6. Statistical Analysis

The samples were described using descriptive statistics, including counts and percentages. For survey items using Likert scales, we collapsed the response options to create dichotomous variables (i.e., Strongly agree/Agree vs. Neither agree nor disagree/Disagree/Strongly disagree and Always/Almost always/Usually vs. Sometimes/Rarely/Never). We used Chi-square and, when indicated, Fisher’s exact tests to look for differences in providers’ knowledge, attitudes, and behaviors by survey sample. Lastly, adjusted logistic regression was used to test for differences in behavior, controlling for survey sample and provider characteristics (gender and years of practice). Findings were considered statistically significant at *p* < 0.05. All statistical analyses were conducted using Stata 16.

## 3. Results

### Participant Characteristics

In the national survey of pediatric oncologists, a total of 195 responses were received, representing a response rate of 15%. For the Survivor HPV Program survey, 39 out of 88 providers completed the survey, representing a response rate of 44%. While most participants were female (56% in the national survey and 79% in the Survivor HPV Program survey), the national survey sample had a higher proportion of males (*p* = 0.027). There were no significant differences in years of practice across the two survey groups. Participant characteristics are summarized in Table 1.

We found statistically significant differences in unadjusted comparisons between the national sample of pediatric oncologists and pediatric oncology providers who participated in our Survivor HPV Program across all measures of HPV vaccine knowledge, attitudes, self-efficacy, and vaccine practices, as shown in Table 2. In terms of knowledge, almost all pediatric oncology providers from the Survivor HPV Program survey (96%) agreed that CCS are at a greater risk than the general population to develop HPV-related malignancies, compared with 38% of the providers in the national sample (*p* < 0.001). Oncology providers in the Survivor HPV Program sample were much less likely than oncologists in the national sample to agree that it should be pediatricians rather than oncologists who recommend (4% vs. 29%, *p* = 0.006) and administer (4% vs. 50%, *p* < 0.001) the HPV vaccine. As a sensitivity analysis, we also looked at the mean Likert scores for each of these variables and found the same pattern of significant differences (Table A1).

All providers who participated in the Survivor HPV Program (100%) agreed with the statement that they know how to recommend the HPV vaccine in a way that leads to vaccination and that they feel confident in addressing parents’ concerns when discussing the HPV vaccine, compared with 66% and 69% of the providers in the national survey, respectively (*p <* 0.001). In comparison with slightly over half (53%) of the providers in the national survey, the majority of Survivor HPV Program survey respondents (88%) believe they know enough about the HPV vaccine (*p* = 0.001). Similarly, in the Survivor HPV Program survey, nearly two-thirds (63%) ‘always’ or ‘almost always’ recommend and discuss the HPV vaccine, a practice reported by only 35% of the providers in the national sample (*p* = 0.013). In logistic regression models controlling for provider sex and years in practice, these associations remained statistically significant (Table 3).

## 4. Discussion

Since the human papillomavirus (HPV) vaccine was approved in the United States in 2006, an estimated 88% of infections with oncogenic HPV subtypes linked to genital warts and most HPV-related cancers have been prevented in teenage girls and young women [19]. Provider recommendation of the HPV vaccine is one of the most important predictors of its uptake [20,21,22]. Increasing the frequency of HPV vaccine recommendations for eligible patients is clearly important; however, the strength of provider recommendations is also paramount [23,24]. This is particularly important among childhood cancer survivors (CCS) who have higher risks of HPV-related cancers and yet are undervaccinated due to a lack of a provider recommendation.

Our finding of low self-efficacy among pediatric oncologists in the national survey for recommending HPV vaccination highlights the need for effective provider training in this setting. According to McRee et al. [25], healthcare providers who feel less confident in addressing parental concerns about the HPV vaccine are less likely to recommend it. Cunningham-Erves et al. [26] have also found self-efficacy to be one of the most important determinants that must be targeted by provider interventions to effectively address parental HPV vaccine hesitancy. Furthermore, Osaghae et al. [27] discovered that HPV-vaccine-hesitant parents were three times more likely to accept the vaccine when counseled by healthcare providers with higher self-efficacy. We found that self-efficacy was significantly higher among our sample of providers who participated in a relatively brief educational intervention, thus providing evidence that self-efficacy to recommend the HPV vaccine is modifiable and an important target for intervention when trying to improve HPV vaccination rates.

Our nationwide survey of pediatric oncologists also revealed persistent misconceptions about the importance of HPV vaccination for CCS. Moreover, there seemed to be low enthusiasm about oncologists recommending and administering the vaccine to this high-risk group. These could be significant barriers to recommendation behaviors and the strength of recommendations by providers, as stated by Btoush et al. [28]. Again, we found that there were significantly fewer misconceptions among the group who participated in the HPV Survivor Program, indicating that knowledge can be improved through an educational intervention with providers.

Importantly, we found significant differences between the nationwide survey of providers and those who participated in our HPV vaccine education program, indicating that the program was effective at increasing HPV vaccine knowledge, attitudes, self-efficacy, and self-reported recommendation practices. According to Leung et al. [29], increasing vaccine initiation and completion rates requires educating clinicians to improve their knowledge about the HPV vaccine as well as their self-efficacy in recommending it. Moreover, providers’ attitudes towards the HPV vaccine have shown to be an important modifiable determinant of their adherence to guidelines and intention to administer the vaccine that should be targeted in vaccination–promotion interventions [30,31]. Our findings highlight the potential efficacy of a 90 min educational program in improving these important determinants, which are essential to increase HPV vaccination rates in this vulnerable population.

Within the CCS demographic, one must additionally address the unique needs and increased vulnerability to secondary malignancies linked to HPV. According to Berkowitz, Rodriguez, and Saraya [32] and Berenson et al. [33], the recommendation of the HPV vaccine is correlated with provider knowledge. A systematic review by Kong et al. [24] also discovered that pediatric healthcare providers with higher HPV-related knowledge are better equipped to deliver superior-quality recommendations. Previous research [9] has also shown that CCS prefer to receive such recommendations from their oncology care team. It is, therefore, reasonable to predict that better recommendations and increased vaccination coverage will result from oncology providers being educated about the HPV vaccine’s benefits in preventing cancer. Given that providers who participated in our program showed increased knowledge about CCS being at greater risk for HPV-related secondary malignancies and felt more confident in recommending the vaccine compared with the national sample, our findings are consistent with this. Furthermore, our results accounting for years of practice support Kong et al.’s [24] recommendation to implement communication training for providers at all stages of their careers.

Additionally, while our study focused on the impact of a provider’s educational training, vaccine practices are also impacted by broader influences, including national, state, and institutional policies. Multilevel interventions and evaluations are warranted to best improve vaccination outcomes for this vulnerable population.

The Survivor HPV Program survey found that a higher percentage of providers recommended the HPV vaccine vs. flu and meningococcal vaccines, highlighting the significance of enhancing providers’ communication skills so that they can make effective recommendations. This was consistent with a recent study by Caldwell et al. [34], who discovered a similar effect when comparing HPV with Tdap and meningococcal vaccination rates among adolescents who received a recommendation against those who did not.

### Limitations

While our study has strengths in being the first to assess HPV vaccine knowledge, attitudes, and practices among pediatric oncology providers, the results should be considered in light of several limitations. The low response rate in the national sample of pediatric oncologists may limit our findings’ generalizability, potentially introducing response bias, as the views and experiences of nonrespondents could differ significantly from respondents. However, providers who responded to the national survey are more likely to be interested in the topic, so our findings may overestimate the knowledge and self-efficacy of oncology providers nationally regarding the HPV vaccine, and thus underestimate the differences between the national group and those who received our training. Other limitations that limit the generalizability of our findings include the cross-sectional design and providers’ self-reported data, which may be prone to recall bias and social desirability bias, particularly when it comes to clinical practices and self-efficacy measures. Furthermore, the national survey was conducted in 2019, and the Survivor HPV Program survey was conducted in 2023. Thus, differences in provider attitudes, knowledge, and practice may reflect overall changes nationally, including the influences of the COVID-19 pandemic, and not only be solely attributable to the provider training. The nature of our study, including a lack of a control group, limits our ability to assess the impact of the COVID-19 pandemic on provider attitudes and practice regarding the HPV vaccine. However, several other studies have explored differences in HPV vaccine attitudes and practices before and after the pandemic and have found that provider attitudes and recommendations of the HPV vaccine [35] and parental intention to vaccinate their adolescent children against HPV [36,37,38] remained unchanged during the COVID-19 pandemic.

Moving forward, we suggest a number of strategies for addressing the aforementioned limitations and improving future investigations. First, future studies should take steps to improve response rates, such as optimizing survey schedules or gaining the support of key stakeholders or professional networks to promote the study and encourage participation. An alternate study design, such as having a control group who did not participate in the Survivor HPV Program, as well as validating self-reported data by using objective measures such as the number of HPV vaccination recommendations, or vaccination rates, may allow for more rigorous comparisons and conclusions. Additionally, controlling for external factors, such as institutional policies, may aid in isolating the impact of the variables under study. Finally, considering the unprecedented impact of the COVID-19 pandemic on healthcare services and practices, future research should look at the potential effects on study outcomes. Despite these limitations, our study provides a unique comparison of HPV knowledge, attitudes, and practice in the care of a vulnerable and understudied group.

## 5. Conclusions

Addressing the disparity in HPV vaccination uptake among CCS is critical to ensuring that they receive the necessary protection against HPV-related malignancies, as recommended by the National Comprehensive Cancer Network (NCCN) and endorsed by the Children’s Oncology Group (COG). Effective strategies to accomplish this goal include educating oncology providers on the greater HPV cancer risk of CCS, improving their self-efficacy in recommending the HPV vaccine, and promoting vaccination in the oncology setting. Our study provides evidence that a short educational program for oncology providers is effective at increasing knowledge of CCS-specific HPV vaccine risks, improving their self-efficacy to recommend the HPV vaccine, and changing practice behaviors.

## Figures and Tables

**Table 1:** Sociodemographic Characteristics of Study Participants by Survey.

Characteristic	National Survey (n = 195)		Survivor HPV Program (n = 24)		
	n	(%) ^+^	n	(%) ^+^	*p*-Value *
**Sex**					
Female	105	(55.6%)	19	(79.2%)	
Male	84	(44.4%)	5	(20.8%)	**0.027**
**Years in Practice**					
0–5 years	25	(13%)	4	(16.7%)	
6–10 years	46	(24%)	6	(25%)	
11–20 years	69	(35.9%)	6	(25%)	
20+ years	52	(27.1%)	8	(33.3%)	**0.740**

* *p*-values based on Fisher’s exact tests. ^+^ Percentages based on the number of nonmissing responses.

**Table 2:** Unadjusted comparison of providers’ knowledge, attitudes, self-efficacy, and HPV vaccine practices.

	National Survey	Survivor HPV Program	
	% Strongly Agree/Agree		*p*-Value *
**Knowledge**			
Childhood cancer survivors are at greater risk of developing HPV-related malignancies than the general population.	38.1	95.8	<0.001
**Attitudes**			
HPV vaccine recommendation should be handled by the general pediatrician rather than by the oncologist.	29	4.2	0.006
HPV vaccine administration should be handled by the general pediatrician rather than by the oncologist.	49.7	4.2	<0.001
**Self-efficacy**			
I know how to recommend the HPV vaccine in a way that leads to vaccination.	65.6	100	<0.001
When discussing the HPV vaccine, I feel confident that I can address parents’ concerns.	69.3	100	<0.001
I feel I know enough about HPV vaccines.	52.7	87.5	0.001
**HPV Vaccine Practices**	**% Always/Almost Always**		***p*** -value *
In your practice, how often do you discuss or recommend the HPV vaccine for age-eligible cancer survivors (regardless of the availability of on-site administration?)	36.4	62.5	0.013

* *p*-values based on Fisher’s exact tests.

**Table 3:** Logistic regression models comparing providers’ knowledge, attitudes, self-efficacy, and HPV vaccine practices by survey administration.

	Odds Ratio *	95% Confidence Interval
**Knowledge**		
Childhood cancer survivors are at greater risk of developing HPV-related malignancies than the general population.	37.35	(4.91–283.95)
**Attitudes**		
HPV vaccine recommendation should be handled by the general pediatrician rather than by the oncologist.	0.10	(0.01–0.79)
HPV vaccine administration should be handled by the general pediatrician rather than by the oncologist.	0.04	(0.01–0.32)
**Self-efficacy**	**	**
I know how to recommend the HPV vaccine in a way that leads to vaccination.	**	**
When discussing the HPV vaccine, I feel confident that I can address parents’ concerns.		
I feel I know enough about HPV vaccines.	5.99	(1.71–21.00)
**HPV Vaccine Practices**		
In your practice, how often do you discuss or recommend the HPV vaccine for age-eligible cancer survivors (regardless of the availability of on-site administration?)	2.84	(1.10–7.32)

* This table represents five logistic regression models (one for each row) with the national survey as the reference group. Each model controls for provider sex and years in practice. ** We could not run logistic regression models for these two variables because 100% of the Survivor HPV Program participants agreed; thus, there was no variance.

## Data Availability

The data that support the findings of this study are available from the corresponding author, L.A.S., upon reasonable request.

## Appendix A. Common Survey Items Used in Both Survey Groups

*Screener Question (only asked in national survey):*

Are you currently in practice as a pediatric oncology/hematology provider?

□ Yes
□ No

If Yes, Please continue with the survey

If No, you are not eligible to participate in this survey, thank you for your time!

**Please answer the following questions. There are no right or wrong answers; we want to know your personal options and experiences as a practicing provider.**

*General Characteristics*

1. Gender

□ Female
□ Male
□ Prefer to self-describe:_____________________
□ Prefer not to say

2. Years in pediatric oncology/hematology practice

□ 0–5
□ 6–10
□ 11–20
□ 20+

*Vaccine Practices*

3. In your practice, how often do you **discuss or recommend** any of the following vaccinations for age-eligible cancer patients or survivors (regardless of availability of on-site administration)?

**Vaccine Type**	**Always/Almost Always**	**Usually**	**Sometimes**	**Rarely**	**Never**
Flu Vaccine	□	□	□	□	□
Meningococcal	□	□	□	□	□
Human Papilloma Virus (HPV)	□	□	□	□	□
Tdap	□	□	□	□	□

*HPV Infection and Vaccine Knowledge*

**Please indicate your agreement with the following statements.**

	Strongly disagree	Disagree	Neither agree nor disagree	Agree	Strongly agree
4. Childhood cancer survivors are at greater risk of developing HPV-related malignancies than the general population.	□	□	□	□	□
5. HPV vaccine is effective.	□	□	□	□	□
6. Most parents/patients think HPV vaccination is important for their child/themselves.	□	□	□	□	□
7. A provider’s recommendation greatly increases HPV vaccination.	□	□	□	□	□
8. I feel comfortable making HPV vaccine recommendations.	□	□	□	□	□
9. I know how to recommend HPV vaccine in a way that leads to vaccination.	□	□	□	□	□
10. When discussing HPV vaccine, I feel confident addressing parents’ concerns.	□	□	□	□	□
11. HPV vaccine recommendation should be handled by the general pediatrician rather than by the oncologist.	□	□	□	□	□
12. HPV vaccine administration should be handled by the general pediatrician rather than by the oncologist.	□	□	□	□	□

**Please indicate your agreement with the following statements.**

	**Agree**	**Disagree**	**Don’t know/not sure**
13. I feel I know enough about HPV vaccines.	□	□	□

Thank you for your participation.

Within the next week, you will receive an email with a link to your electronic gift card. If you have any questions you can contact the study PI at laura.aubree.shay@uth.tmc.edu.

**Table A1:** Unadjusted comparison of mean scores of providers’ knowledge, attitudes, self-efficacy and HPV vaccine practices.

	National Survey	Survivor HPV Program	*p*-Value *
	**Mean Scores ****		
**Knowledge**			
Childhood cancer survivors are at greater risk of developing HPV-related malignancies than the general population.	3.37	4.5	<0.001
**Attitudes**			
HPV vaccine recommendation should be handled by the general pediatrician rather than by the oncologist.	2.91	2.41	0.028
HPV vaccine administration should be handled by the general pediatrician rather than by the oncologist.	3.46	2.45	<0.001
**Self-efficacy**			
I know how to recommend the HPV vaccine in a way that leads to vaccination.	3.77	4.5	<0.001
When discussing the HPV vaccine, I feel confident that I can address parents’ concerns.	3.87	4.42	0.006
I feel I know enough about HPV vaccines. ***	1.59	1.17	0.004
**HPV Vaccine Practices**	**Mean Scores**		
In your practice, how often do you discuss or recommend the HPV vaccine for age-eligible cancer survivors (regardless of the availability of on-site administration?)	2.29	1.42	<0.001

* *p*-values based on independent sample t-tests. ** Knowledge, attitudes, and self-efficacy items (except the one noted below) were based on a 5-point Likert scale with 1 indicating strongly disagree and 5 indicating strongly agree. Responses to The HPV Vaccine Practice were on a 5-point Likert scale with 1 indicating always/almost always and 5 indicating never. *** This item included a 3-point Likert scale for responses with 1 = agree, 2 = disagree, and 3 = don’t know/not sure.
